# ANALYSIS OF THE EFFECTS OF SOCIAL CAPITAL ON SMOKING BEHAVIOR AMONG OLDER ADULTS IN CHINA

**DOI:** 10.1093/geroni/igz038.3290

**Published:** 2019-11-08

**Authors:** Xiaojie Sun, Linlin Yuan

**Affiliations:** 1 Shandong University, Jinan, Shandong, China; 2 Shandong Maternal and Child Health Hospital, Jinan, China

## Abstract

By 2030, China will become the most aging country in the world. At the same time, China is the world's largest producer and consumer of tobacco, also the largest victim of tobacco. Tobacco exposure is one of the most important risk factors for many chronic non-communicable diseases among older adults. Based on the data from China Health Retirement Longitudinal Study(2011&2015), we aim to analyze the effects of base-period social capital on current smoking behaviors among Chinese older adults of 60 and above (N=7686) with univariate analysis and ordered logit model. Resluts show that, older adults with high social trust (OR=0.783) preferred to choose not to smoke ; those who had emotional support (OR=0.933) and financial support (OR=0.967) would be more possible to choose not to smoke; and older adults were more likely to choose heavy smoking if they had two or more types of social participation (OR=1.223). This study demonstrates that social trust and social support are the protective factors of smoking behavior among Chinese older adults, while social participation was a risk factor. The Chinese government has launched "the 2030 Plan for healthy China" to promote people’s healthy behaviors, and this study will provide good evidence for actions aiming at reducing smoking behaviors among Chinese older adults.

